# Expanding the Map of Intrinsic and Extrinsic Aspirations Using Network Analysis and Multidimensional Scaling: Examining Four New Aspirations

**DOI:** 10.3389/fpsyg.2019.02174

**Published:** 2019-10-11

**Authors:** Frank Martela, Emma L. Bradshaw, Richard M. Ryan

**Affiliations:** ^1^Department of Industrial Engineering and Management, Aalto University, Otakaari, Finland; ^2^School of Business, Aalto University, Otakaari, Finland; ^3^Institute for Positive Psychology and Education, Australian Catholic University, North Sydney, NSW, Australia; ^4^Department of Psychology, University of Rochester, Rochester, NY, United States

**Keywords:** goals, goal content theory, intrinsic goals, life aspirations, self-determination theory

## Abstract

Life goals or aspirations can be distinguished as intrinsic or extrinsic, with different implications for well-being. In this study we used network analysis to reexamine this intrinsic-extrinsic distinction, illustrating how novel candidate aspirations can be mapped along this dimension using innovative methods. We identify four previously unexamined life aspirations, predicting that aspiring for *power* and *social adherence* would group with extrinsic goals, whereas aspiring for *self-expression* and *mastery* would group with intrinsic goals. In two samples (*n* = 196; *n* = 346) we analyze both the original life-goal variables and these novel aspirations using factor analysis, multidimensional scaling, circumplex modeling, and a network analysis using a graphical least absolute shrinkage and selection operator (glasso) to estimate the network structure of aspirations using EBIC. Replicating past intrinsic-extrinsic distinctions, all four methods further classified power and social adherence as extrinsic goals, and self-expression and mastery as intrinsic goals. The intrinsic-extrinsic distinction was also clearly visible in the network analysis. Finally, consistent with hypotheses, power and social adherence were unrelated to wellness indicators, whereas self-expression and mastery correlated positively with well-being indicators and negatively with ill-being indicators, supporting their hypothesized nature as extrinsic and intrinsic, respectively.

## Introduction

People typically have various life aspirations or long-term personal goals that lead them to make certain choices and engage in particular lifestyles. Within research on such life goals, one important distinction has been between *intrinsic* and *extrinsic aspirations* (Kasser and Ryan, 1993, 1996), which have been empirically explored within *self-determination theory* (SDT; Ryan and Deci, 2017). *Intrinsic aspirations* are defined as those life goals thought to be congruent with, and relatively directly satisfying of, people’s basic psychological needs. According to SDT, pursuing intrinsic aspirations should enhance various wellness-related outcomes. In contrast, *extrinsic aspirations* focus on instrumental outcomes such as image, fame, or money, which, because they do not directly satisfy basic psychological needs, are thought to have a neutral or even a negative impact on wellness, often by crowding out or interfering with the more direct pursuit of intrinsic goals (e.g., Unanue et al., 2014).

A broad stream of research on intrinsic and extrinsic aspirations (reviewed in Ryan and Deci, 2017) has indeed shown that both *having* intrinsic aspirations and *attaining* them is associated with various indicators of well-being, whereas having and attaining extrinsic aspirations are not connected to well-being, and in some cases, are connected to indicators of ill-being (e.g., Kasser and Ryan, 1993, 1996; Niemiec et al., 2009; Romero et al., 2012; Bradshaw et al., 2018a). Such results have been replicated across a wide variety of cultures (e.g., Ryan et al., 1999; Martos and Kopp, 2012) and in various populations from high school students (Lekes et al., 2010; Nishimura et al., 2019) to older adults (Van Hiel and Vansteenkiste, 2009). Thus, in contrast to some goal theories which suggest that attainment of any valued goal is beneficial for psychological health (e.g., Locke and Latham, 1990), it seems that “not all goals are created equal,” in that aspiring for and even attaining certain types of goals may not lead to the happiness and wellness often anticipated.

The intrinsic aspirations examined in past studies typically include *affiliation* (having deep, enduring relationships), *community contribution* (generativity and helping others), *personal growth* (self-acceptance and development), and *physical health*, whereas typical extrinsic aspirations have included *financial success* and wealth, *popularity* (being famous or admired), and having an *appealing image* (being physically attractive) (e.g., Kasser and Ryan, 1996; Niemiec et al., 2009). These seven specific aspirations are most often located within their respective higher-order intrinsic and extrinsic domains using factor analysis (Kasser and Ryan, 1996; SabzehAra et al., 2014). However, such analyses preclude a more fine-grain discussion about how the specific aspirations are associated with each other, both within and between the higher-order categories. That is, beyond their membership in a higher order factor, more nuanced relationships between these variables is neglected.

To address the limited scope of the factor analytic approach, Grouzet et al. (2005) explored the dimensional nature of aspirations using multidimensional scaling and circular stochastic modeling. The resultant circumplex model of aspirations placed ten aspirations around the circumference of a circle, comprised of two axes. The first axis depicted the intrinsic vs. extrinsic dimension, and the orthogonal axis represented a physical self-focused vs. self-transcendent dimension. On the circumplex, community contribution aspirations were, for example, on the intrinsic end of the intrinsic/extrinsic axis and were more self-transcendent than physical self-focused. However, some of the aspirations analyzed by Grouzet et al. (2005) did not fit neatly on the intrinsic/extrinsic dimension. Specifically, spirituality and hedonism goals lacked clear intrinsic or extrinsic quality, arguably because goals of both types could be either intrinsically or extrinsically motivated (e.g., see Ryan et al., 1993).

Although the circumplex model provided an important visual demonstration of the dimensional nature of the aspirations, it did not give detailed information about the strength and direction of the links between specific aspirations or about the interconnectedness or influence of specific aspirations. For example, although two aspirations may appear distal on the circumplex, the model does not give direct information about whether the correlation between those two aspirations is positive, negative, or non-significant. Indeed, while Grouzet et al. (2005) made deductions about the associations between pairs of aspirations based on their distance from each other on the circumplex, they acknowledged that such claims were speculative. In addition, as the focus of Grouzet et al. (2005) was on the dimensional modeling, they did not examine relations between aspiration variables and well-being or ill-being outcomes. Thus, among our aims in the present study, we want to replicate and extend the dimensional analyses by Grouzet et al. (2005).

To address the above-mentioned gaps in prior research, we seek to further expand these analyses through the novel use of network analysis. Network analysis provides an alternative analytic approach to examining the connections between nodes in a network (i.e., in this case, between specific aspirations) while controlling for all the other nodes in the network. Thus, network analysis provides a novel test of (a) how individual aspirations relate to each other; and (b) whether the distinction between intrinsic and extrinsic aspirations holds even when it is not prespecified in the model.

Finally, in the context of these methodological advances, we seek to expand the map of aspirations by testing the intrinsic or extrinsic qualities, and predictive utility, of four candidate aspirations: power, social adherence, self-expression, and mastery. We are interested in these four aspirations because, although each has been previously studied in their own right, they have not been examined in relation to intrinsic/extrinsic distinction. Integrating these four goals into the literature on intrinsic/extrinsic goals will both inform research on these goals in their own right and illustrate how the aspiration space covered by the goal content theory has room for additional candidate life goals. More generally, a deeper understanding of the relations between various life goals and their links with wellness-relevant outcomes requires replication of existing results, methodological and substantive expansion, and, ideally, a multi-method approach. Our proposed combination of existing and innovative methodologies in conjunction with our expansion of intrinsic and extrinsic aspirations thus gives our study methodological and substantive import, and potentially provides a roadmap for others who might wish to explore additional candidate life aspirations in future studies.

## Power, Social Adherence, Self-Expression, and Mastery as Four Previously Unexamined Aspirations

In this study we examine the set of aspirations that has been traditionally examined (namely, financial success, fame, and image as extrinsic goals; and personal growth, health, close relationships, and community as intrinsic goals) alongside four new candidate aspirations: power, social adherence, self-expression, and mastery. We predict, for reasons explained below, that aspirations for power and social adherence will exhibit the qualities of extrinsic aspirations, and that the goals of self-expression and mastery will exhibit the qualities of intrinsic aspirations.

The aspiration for *power* has been argued to be one of the most important human social motives by several theorists (e.g., McClelland, 1985; Winter et al., 1998), and its influence on various behavioral and cognitive processes has been widely examined (e.g., Fodor and Smith, 1982). Following Winter et al. (1998) we recognize that power can represent both the aspiration to control or dominate over others, or to influence and have an impact on other people. Herein we focus primarily on the dominance and control aspects of power. Some research has investigated how power motivation, when inhibited, is associated with high blood pressure and other signs of illness (see McClelland, 1979), and research on power as a value has shown it to be negatively associated with life satisfaction and positively associated with depression (Bobowik et al., 2011; Sortheix and Schwartz, 2017). However, previous research has not examined whether power as a life goal, especially a focus on controlling others, would exhibit the characteristics of intrinsic or extrinsic goals. Our hypothesis is that such aspirations for power should align with the extrinsic goals. Extrinsic aspirations are typically dependent on external contingencies, and thus we expect power to be a highly extrinsic aspiration. Like wealth, popularity, and image aspirations, the perception of one’s power depends on external inputs, other people need to be dominated for one to experience themselves as having power over others. Power as a value has also been grouped together with self-protective, compensatory, and deficit-oriented values, attesting to its extrinsic nature (Sagiv and Schwartz, 2000; Sortheix and Schwartz, 2017). Furthermore, motives for power have been interpreted as a compensation motivation for having previously been deprived of feelings of autonomy (Ryan and Deci, 2017, p. 90). For example, Lammers et al. (2016) recently demonstrated in multiple experiments that gaining autonomy dampens participants’ desire for power, suggesting its compensatory nature. To our knowledge, the hypothesis that power is an extrinsic goal has been tested in only one previous study (Ryan et al., 1999), although neither its factor loadings nor its univariate correlates with well-being and ill-being indicators were reported therein. Given the salience of power motivations in domestic, work, and political life, understanding its relations with other aspirations is especially important.

A second potential extrinsic goal we explore is that of *social adherence*. Being included in a social group and avoiding social exclusion are powerful motives for human beings (Williams, 2009). Social exclusion has been shown to be highly aversive for individuals (e.g., Eisenberger et al., 2003; Legate et al., 2013), and humans sometimes go to great lengths to fit into the group (e.g., Asch, 1956). As social animals, human survival in hunter-gather settings was intimately tied to group membership, making exclusion from the group a life-threatening risk (Boehm, 2001; Wesselmann et al., 2012). This evolutionary pressure to avoid social exclusion has arguably made humans highly sensitive to the threat of being ostracized and motivated to ensure the continual fitting in into the group (Spoor and Williams, 2007; Kerr and Levine, 2008). Accordingly, humans can be expected to develop a defensive and extrinsic desire for *social adherence* as the goal to make sure one “fits in” and is not excluded from the group^1^. Thus, we want to examine the hypothesis that such desires for social adherence represent an extrinsic goal. In contrast, affiliation represents a positive side of relatedness and has traditionally aligned with intrinsic goals (Kasser and Ryan, 1996).

We also examine two novel intrinsic goals. Intrinsic goals are thought to satisfy the basic psychological needs for autonomy, competence, and relatedness (Deci and Ryan, 2000), and satisfaction of these three needs has been shown to mediate the positive correlations between intrinsic aspirations and well-being (e.g., Niemiec et al., 2009). Therefore, it would be interesting to examine goals that are especially prone to lead to the satisfaction of a particular need. In this regard, we would expect that aspiring to be *self-expressive*, or to pursue authentic and volitional interests and passions, would particularly yield autonomy satisfaction, as self-expression would lead to behavior that the person would perceive as volitional and emanating from one’s self. Past research has shown that self-direction as a value is associated with affective and psychological well-being (Sagiv and Schwartz, 2000; Joshanloo and Ghaedi, 2009).

Similarly, those aspiring to *mastery*, defined as seeking to develop high-level skills and capabilities, would likely experience especially competence but also autonomy need satisfactions. Previous research has shown that mastery-orientation is associated with academic well-being in educational settings (e.g., Daniels et al., 2008; Tuominen-Soini et al., 2012). However, research thus far has not examined either self-expression or mastery as personal life goals, nor how such goals would align with intrinsic and extrinsic categories. Accordingly, in the present research we construct measures of *self-expression* and *mastery* as life aspirations and examine whether they demonstrate the psychometric characteristics of intrinsic life goals, and whether they show positive relations to well-being indicators.

In addition to testing these four new aspirations, we also examined, in a more exploratory manner three additional goals – hedonism, spirituality and safety – that have been included in some past studies of aspirations (e.g., Grouzet et al., 2005) and that have more complex relations with intrinsic/extrinsic goal dimensions and with need satisfaction. Hedonism, for example, takes many forms, some growth-oriented and focused on intrinsic satisfactions in experience (see Bilsky and Schwartz, 1994; Sortheix and Schwartz, 2017), others on over-indulgent pleasure and escapism. Spirituality, similarly, can have both intrinsic and extrinsic aspects (Ryan et al., 1993). Fitting with this, Grouzet et al. (2005) found that spirituality does not fit neatly within either intrinsic or extrinsic aspirations. However, past research on goals has demonstrated that they can be organized in a circumplex with two primary axis: Intrinsic vs. extrinsic and self-transcendence vs. physical self (Grouzet et al., 2005). And while hedonism and spirituality are ambiguous as regards their location on the intrinsic-extrinsic axis, spirituality emerges clearly as a self-transcendent goal and hedonism emerges clearly as a goal related to the physical self. Given our interest in the circumplex model, we thus wanted to include these two goals that tend to offer the opposing examples as regards the other axis. Finally, safety appears to be a more intrinsic goal. However, within SDT safety has been discussed as a *deficit need* (Ryan and Deci, 2000, 2017; Martela and Ryan, 2019), something that becomes salient primarily under conditions of threat or insecurity. Thus, unlike other intrinsic goals, safety is not a growth-focused life goal, but a protective motive, and thus is not expected to be associated with wellness indicators. An additional novelty of our study is not only the examination of how these intrinsic and extrinsic goals *taken together* relate to well-being, which has been the typical approach in the literature, but to look at each intrinsic and extrinsic goal separately in relation to indicators of well-being and ill-being.

## The Present Study

In the present study, we measured people’s life aspirations to examine which goals had the characteristics of extrinsic goals and which had the characteristics of intrinsic goals. For this purpose we examined the four novel goals of self-expression, mastery, power, and social adherence along with nine previously examined goals from Grouzet et al. (2005): Affiliation, community contribution, physical health, safety, money, popularity, image, hedonism, and spirituality. We excluded two of the goals used by Grouzet et al. – conformity and self-acceptance – due to conceptual overlap. Conformity, as noted, bears similarity to social adherence but the former also referred to elements of blending in with others, in the sense of having similar tastes, which we wanted to exclude from our social adherence construct to make it conceptually clearer. Self-acceptance, in turn, has been studied in past research as an intrinsic goal (Kasser and Ryan, 1993; Grouzet et al., 2005) but we decided to exclude it because its definition and operationalizations included elements of both self-expression (evident in items such as “I will feel free”) and mastery (evident in items such as “I will feel good about my abilities”) and thus – given our interest in self-expression and mastery as two separate goals – the conceptual overlap would have distorted the results.

In addition to replicating the previously established intrinsic nature of affiliation, community contribution, physical health, and safety, the extrinsic quality of aspirations for money, popularity, and image, the self-transcendent quality of spirituality, and the physical self–related quality of hedonism, we wanted to examine the following novel hypotheses:

H1: Self-expression and mastery will be grouped together with other intrinsic aspirations.
H2: Power and social adherence will be grouped together with other extrinsic aspirations.

These hypotheses were tested in four ways. First, we used classical exploratory factor analysis which has most commonly been used in the intrinsic and extrinsic aspirations literature (e.g., Kasser and Ryan, 1996). Second, we used multidimensional scaling which represents the goals in a two-dimensional space where one axis has previously been shown to represent the intrinsic/extrinsic dichotomy and the other axis includes physical self vs. self-transcendence (Grouzet et al., 2005). This allowed us to see how mutually compatible the various aspirations are and whether our newly introduced aspirations organize meaningfully within this two-dimensional space – as well as providing an opportunity to test whether the two-dimensional structuring of goals found in Grouzet et al. (2005) would replicate in these new samples. Third, we wanted to examine and confirm whether the aspirations adhered to a circumplex structure by using Browne’s (1992) circular stochastic modeling approach.

Finally, in the second sample, we complemented these existing analytic strategies with network analysis. By providing an analytically different way to examine the connections between studied variables we reveal new information about how the goals are related to each other which could not be demonstrated by traditional methods. We used a graphical least absolute shrinkage and selection operator (glasso) to estimate the network structure of aspirations using the extended Bayesian information criterion (EBIC). This allowed us to examine partial correlations between each possible pair of aspirations, controlling for all the other aspirations in the network (Bell and O’Driscoll, 2018). Contrary to conventional partial correlations, which assess the link between two variables controlling for other covariates, the glasso uses a regularizing weighted penalty to favor nodes (in this case, aspirations) with univariate strength (Epskamp et al., 2018). The regularization strategy reduces spurious partial correlations (“edges,” in the network analysis context) to zero, resulting in a sparse network with minimal redundancy. The benefit of including network analysis in conjunction with more traditional methods is that it is not model-bound, and the results are generated entirely by the data (Bell and O’Driscoll, 2018). If a glasso network of aspirations is similar to that derived using more traditional methods such as factor analysis, multidimensional scaling, and circumplex modeling, it would bolster our confidence about the structure of aspirations as well as provide new information about the influence of specific aspirations in the form of network centrality metrics such as strength, betweenness, and closeness. By examining these metrics for each aspiration, we will be able to discuss the influence of specific aspirations. Also, with the aim to identify intrinsic and extrinsic clusters of aspirations in a novel way, we employed the spinglass algorithm (Bell and O’Driscoll, 2018).

Further, we look at the relations between individual intrinsic and extrinsic goals and well-being, controlling for overall goal importance, with the prediction that the intrinsic goals should have a positive relation to indicators of well-being and a negative relation to indicators of ill-being. For the extrinsic goals we expected neutral or negative relations with well-being indicators, and positive or neutral relations with indicators of ill-being (see e.g., Romero et al., 2012). Our specific hypotheses include:

H3: Self-expression, mastery and other intrinsic goals (affiliation, health, and community contribution) will demonstrate a positive relation to well-being indicators and a negative relation to ill-being indicators.
H4: Power, social adherence and other extrinsic goals (financial success, popularity, image) will demonstrate either a neutral relation or negative relation with well-being indicators and a neutral or positive relation with ill-being indicators.

### Method

#### Participants and Procedure

With the aim of recruiting at least 180 participants, the first sample was gathered through Mturk, which has become a popular and relatively reliable source of study participants in behavioral sciences (Mason and Suri, 2012). For factor analyses, recommendations based on simulations suggest that given a variables-to-factors ratio of 6 to 1, approximately 150 participants are needed for good agreement (Mundfrom et al., 2005). Accordingly, 197 participants from US answered the whole survey, but one was excluded (same answer to all questions), leaving a final sample of 196. To replicate these findings, a second sample was subsequently collected through Mturk. This time we aimed for excellent agreement, which required approximately 300 participants (Mundfrom et al., 2005). We thus recruited 373 participants who answered the whole survey, but based on two inattention check questions added for this study, 32 participants were discarded, resulting in a final sample of 341. This research was carried out in accordance with the recommendations of the Research Ethics Committee of the Aalto University, which according to the national standards didn’t require formal approval for this kind of anonymous survey study. In accordance with the Declaration of Helsinki, we sought informed consent from all study participants by giving them relevant information about the study before they signed up, and they gave their consent anonymously by virtue of agreeing to complete the online form. Of the participants 52.6%/49.9% (first sample/second sample) were male, 46.9%/49.9% female, with one/one preferring not to say. The mean age was 35/36 (range 18/21 to 70/73). They identified as Caucasian (79.6%/79.5%), African American (7.7%/7.9%), Hispanic (5.6%/5.3%), Asian (5.1%/5.6%), and Native American (1.0%/0.6%), with 1%/1.2% preferring not to say. Although the study was not preregistered, we formed all the hypotheses before data collection.

### Measures

#### Life Aspirations

Participants rated various life aspirations based on “how important each goal is to you.” Scales to measure *affiliation* (5 items, α = 0.81/0.87, e.g., “To have people show affection to me, and me to them.”), *community contribution* (3 items, α = 0.85/0.85, e.g., “To do things that will make other people’s lives better.”), *popularity* (3 items, α = 0.80/0.89, e.g., “To be admired by many people”), *financial success* (4 items, α = 0.72/0.78, e.g., “To be financially successful”), *hedonism* (3 items, α = 0.76/0.78, e.g., “To experience a great deal of sensual pleasure”), *image* (5 items, α = 0.89/0.86, e.g., “To have an image that other’s find appealing”), *health* (4 items, α = 0.87/0.87, e.g., “To be in good physical shape”), *spirituality* (5 items, α = 0.94/0.95, e.g., “To live and act in agreement with my religious/spiritual beliefs”), and *safety* (4 items, α = 0.72/0.80, e.g., “To feel safe and secure.”) were taken from Grouzet et al. (2005) *Aspiration Index*, and evaluated on a scale from 1 (not at all important) to 7 (centrally important). The items used to measure *self-expression, mastery, power*, and *social adherence*, were developed in this study and are reported below.

#### Well-Being

To measure *positive and negative affect*, we used the *Scale of Positive and Negative Experience* (SPANE; Diener et al., 2010), which asks the extent to which respondents are experiencing 6 positive (e.g., happy, pleasant; α = 0.93/0.94) and 6 negative (e.g., 6 sad, unpleasant, α = 0.93/0.92) emotions on a scale from 1 (Very rarely or never) to 5 (Very often or Always). *Vitality* was assessed with five items (e.g., “I feel alive and vital.”) from *Subjective Vitality Scale* (SVS; Ryan and Frederick, 1997), rated on a scale from 1 (not at all true) to 7 (very true), α = 0.91/0.92. For *life satisfaction*, the five-item *Satisfaction with Life Scale* (SWLS; Diener et al., 1985) was rated (e.g., “I am satisfied with my life”) on a scale from 1 (strongly disagree) to 7 (strongly agree), α = 0.92/0.94. *Meaning in life* was measured with *Presence of Meaning Scale* (Steger et al., 2006) that includes five items (e.g., “My life has a clear sense of purpose.”) evaluated on a scale from 1 (not at all true) to 7 (very true), α = 0.95/0.95. *Anxiety* was assessed with the *Generalized Anxiety Disorder* 7-item Scale (GAD-7; Spitzer et al., 2006) (e.g., “Feeling nervous, anxious or on edge”), using a scale from 0 (not at all) to 3 (nearly every day), α = 0.93/0.95. *Stress* was assessed with the 10 item version (e.g., “How often have you found that you could not cope with all the things you had to do?”) of the *Perceived Stress Scale* (Cohen and Williamson, 1988) evaluated on a scale from 1 (Never) to 5 (Very often), α = 0.90/0.89.

## Results

### Item Selection and Reliability

Based on the conceptual definitions and review of the relevant literature, a pool of 7 items for self-expression, 8 items for mastery, 7 items for power, and 7 items for social adherence were generated and included in the survey. The items were mixed together with other goals, and participants were asked to evaluate “how important each goal is to you” on a scale from 1 (not at all important) to 7 (centrally important). Our aim was to have a final scale of 4 items for each of the goals, and we proceeded in all cases by first deleting items that were flagged as theoretically incongruent, or had a correlation exceeding 0.80 with another item and thus were deemed redundant (Clark and Watson, 1995). After that, using sample 1, we performed stepwise deletion of the items with the lowest item-total correlation until we reached four items.

Regarding the 7 items for *power*, one item was removed as it correlated.81 with another and was deemed redundant (Clark and Watson, 1995). Stepwise deletion of two items with lowest item-total correlations led to the final scale of four items (e.g., “To have people obey me”) that had a reliability of α = 0.90/0.92. For the 7 *self-expression* items, we first deleted one item (“To feel free”) based on feedback that it is too abstract. After that we deleted stepwise two items to have a final scale of 4 items (e.g., “To be free to do things that really interest me”), with a reliability of α = 0.80/0.81. For the 8 *mastery* items, we deleted two items that we deemed, based on feedback, as theoretically incongruent with the construct. Stepwise deletion of two items led to the final scale of 4 items (e.g., “To set goals and make sure that I achieve them”) that had a reliability of α = 0.81/0.82. Finally, for the seven items for social adherence stepwise deletion of 3 items led to the final scale of four items (e.g., “To be approved by people around me”) with a reliability of α = 0.88/0.89. The final items used to assess power, social adherence, self-expression, and mastery aspirations can be found in Appendix 1.

### Factor Structure

To explore how well the goals load onto the intrinsic and extrinsic factors, we followed best practices for exploratory factor analyses (Osborne et al., 2008) by conducting a principal axis factoring with direct oblimin rotation. Both Kaiser-Meyer-Olkin coefficient (0.837/0.877) and Bartlett’s test of sphericity (*p* > 0.001 in both samples) showed that both samples were adequate for factor analysis. The number of factors to extract was determined using parallel analysis implemented by O’Connor’s (2000) SPSS program and principal components analysis (PCA). In both samples, a two-factor solution was recommended, explaining 61.2%/64.2% of total variance. As shown in Table 1, the hypothesized intrinsic goals – self-expression, mastery, health, safety, and affiliation – loaded clearly on one factor, and the hypothesized extrinsic goals – power, social adherence, popularity, image, and financial success – loaded on the other in both samples. Spirituality and hedonism did not load strongly on either factor, although hedonism tended to have slightly higher loadings on the extrinsic factor. Self-expression and mastery thus loaded together with other intrinsic goals, and power and social adherence together with other extrinsic goals, supporting hypotheses 1 and 2.

**TABLE 1:** Loadings from factor analysis of different life goal scores.

	**Sample 1**		**Sample 2**	
		
	**Factor 1**	**Factor 2**	**Factor 1**	**Factor 2**
Self-expression	**0.809**	–0.018	**0.922**	–0.142
Mastery	**0.680**	0.077	**0.730**	0.124
Physical health	**0.729**	0.048	**0.789**	–0.004
Safety	**0.734**	–0.102	**0.832**	–0.117
Affiliation	**0.803**	–0.020	**0.821**	0.022
Community contribution	**0.617**	0.070	**0.620**	0.136
Power	–0.318	**0.842**	–0.244	**0.911**
Social adherence	0.142	**0.717**	0.188	**0.659**
Popularity	–0.061	**0.930**	–0.037	**0.896**
Image	–0.086	**0.875**	0.040	**0.827**
Financial success	0.189	**0.670**	0.219	**0.605**
Hedonism	0.252	0.546	0.383	0.445
Spirituality	0.165	0.245	0.205	0.149

**Extraction: Principal axis factoring Rotation: Direct oblimin. Loadings above 0.60 in bold.**

Next, to replicate the intrinsic/extrinsic distinction using another method, we examined the potential circumplex structure of the goals that was proposed and supported by the results of Grouzet et al. (2005). For this purpose, we used multidimensional scaling (MDS) utilizing the Proxcal function in SPSS 22. The technique represents the goals as points in a two-dimensional space where the distance between goals reflects their empirical relation to each other. The closer two goals lie to each other, the more consistent or complementary they are with each other, while goals more distant from each other are more in conflict or inconsistent with each other.

**FIGURE 1:**
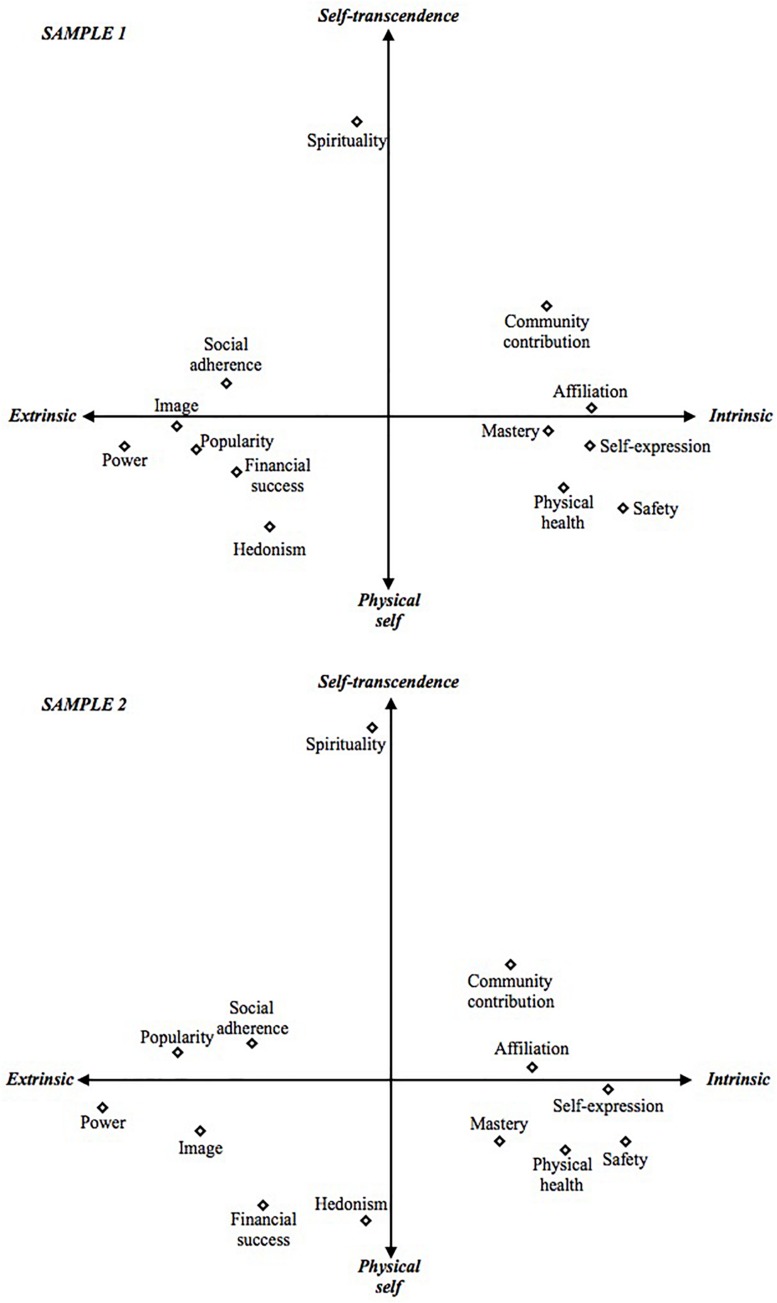
Two-dimensional representation of the goals in both samples resulting from multidimensional scaling analysis of the 13 goals. Stress 1 = 0.0728/0.0695, dispersion accounted for = 0.995/0.995, Tucker’s coefficient of congruence = 0.997/0.998.

Following the procedure of Schwartz et al. (2012), we conducted MDS using ordinal proximity transformations, Euclidian distance measures, Torgerson initial configurations, and Z-score transformations of goal importance. The resulting two-dimensional plots are in Figure 1.

The axis resulting from MDS are not predetermined but derived from the data. Yet, based on an examination of the resulting axis, we can make two conclusions. First, the horizontal dimension conforms well to the distinction between intrinsic and extrinsic goals. All the hypothesized intrinsic goals – self-expression, mastery, affiliation, health, safety, and community contribution – are found on the left side of the circumplex, while all the hypothesized extrinsic goals – power, social adherence, image, popularity, and financial success – are located on the right side of the circumplex. Spirituality lies in the middle, as expected, given that it does not seem to fit well into either intrinsic or extrinsic goals. In sample 2, hedonism is also located in the middle. The analysis thus supports the distinction of goals into intrinsic and extrinsic, and further supports hypotheses 1 and 2. The horizontal axis, in turn, seems to conform to the distinction drawn by Grouzet et al. (2005) between goals related to the physical self vs. self-transcendence. Spirituality, community contribution, social adherence, affiliation, and popularity in sample 2 – which all are characterized by some form of self-transcendence – are located in the top end of the figure, whereas hedonism, safety, and physical health – more clearly related to the physical self – appear as the bottom-most goals in the figure, along with financial success in sample 2. The circumplex thus replicates the analysis by Grouzet et al. (2005), while also extending it to include self-expression, mastery, social adherence, and power as new goals.

**FIGURE 2:**
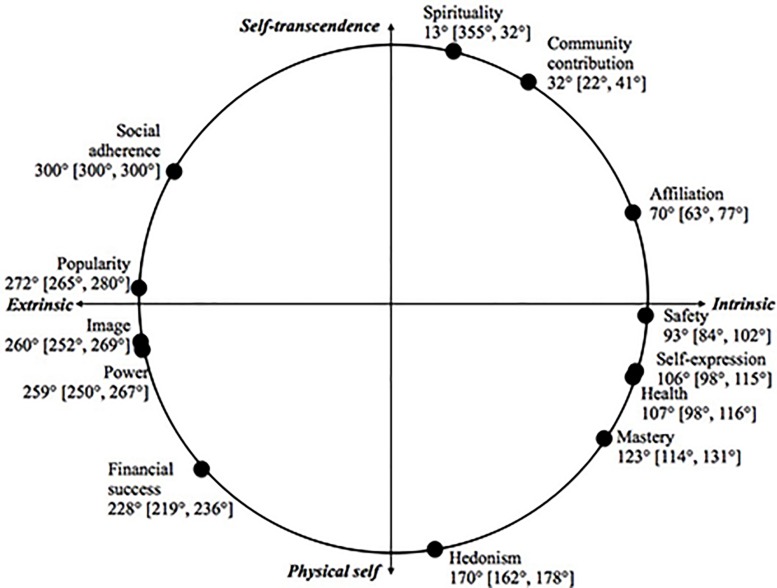
The CircE analysis with point estimates and 95% CIs for polar angles.

While the MDS produced the expected spatial relations between the variables and seemed to adhere to a circumplex ideal, we wanted to test this circumplex structure also using more confirmatory methods. For this purpose, following Grouzet et al. (2005), we used Browne’s (1992) circular stochastic modeling approach, implemented with the CircE package in R that provides results convergent with those obtained by CIRCUM (Grassi et al., 2010). It examines how well the correlation matrix fits a structure where the correlation between two variables is the function of the angle between the variables on the perimeter of a circle. This is thus a more specific hypothesis compared to MDS, which only expects the organization of the variables to reflect a two-dimensional space. The CircE analysis in sample 2 converged after 71 iterations. Most of the fit indices demonstrated good fit (CFI = 0.937, GFI = 0.925, SRMR = 0.069), though the RMSEA was.104 (90% CI: 0.091, 0.118), which could be due to small sample size (Kenny et al., 2015). The estimated item communality indices ranged from 0.51 to 0.90. The point estimates and 95% CIs for polar angles as well as a graphical representation of the resulting circumplex are presented in Figure 2. Evidently, the positions of the aspirations replicate the intrinsic-extrinsic distinction and the self-transcendence-physical self distinction observed in the MDS. These results thus confirm that the nature of the relationships between aspirations can be described as an ordering of variables along the circumference of a circle.

### Network Structure

To complement and extend the results derived using factor analysis and multidimensional scaling, we conducted a cutting-edge examination of the structure of aspirations using network analysis. We first computed a correlation matrix which provides the foundation of the network, and then used the EBICglasso approach in R 3.5.1 (R Core Team, 2018). We carried out our analyses using packages including qgraph (Epskamp et al., 2012), glasso (Friedman et al., 2018), bootnet (Epskamp et al., 2018), dplyr (Wickham et al., 2018), psych (Revelle, 2018), ggplot2 (Wickham, 2009), igraph (Csardi and Nepusz, 2006), and tidyr (Wickham and Henry, 2018).

**FIGURE 3:**
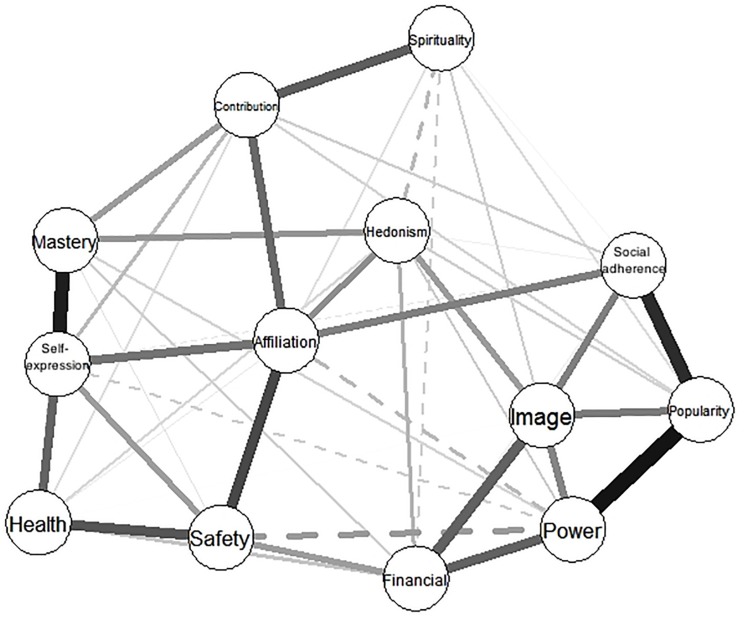
The network structure of intrinsic and extrinsic aspirations derived using the EBICglasso approach. Solid edges (lines between variables) signify positive links between variables, dotted lines are negative links. Edge thickness denotes the strength of the association.

The glasso network structure of aspirations in sample two is shown below in Figure 3. The circles represent each aspiration node, while the edges (the lines) between nodes demonstrate the partial correlations (controlling for all other nodes in the network). The distance between the nodes is intuitive, meaning that nodes more to the left or right of the network are statistically furthest from the nodes on the other side. A solid edge indicates that the link is positive, while dashed edges signify negative links. Edge widths depict the strength of the partial correlations. Per our expectations, and aligned with the results from the factor analysis, multidimensional scaling, and circumplex modeling, Figure 3 shows that the candidate aspirations of self-expression and mastery sit on the left side of the network with strong links to the intrinsic aspirations for health, affiliation, and community contribution. Also, as hypothesized, power and social adherence are to the right of the network with strong links to extrinsic aspirations for financial success, image, and popularity. Notably, the novel aspiration for power has negative links to the novel aspiration for self-expression, as well as to affiliation and safety.

**FIGURE 4:**
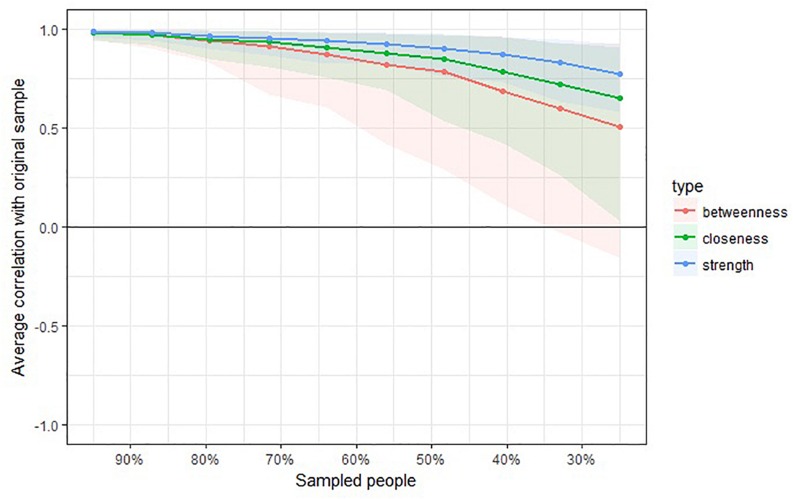
Strength centrality, closeness centrality, and betweenness centrality stability estimates across bootstrapped samples of decreasing size in study 2.

Next, we assessed the centrality of each aspiration using strength, betweenness, and closeness metrics. To ensure robust centrality estimates we used the bootstrapping technique outlined by Bell and O’Driscoll (2018), which involves removing an increasing number of rows from the sample and recalculating the centrality indices to then obtain a correlation stability coefficient (average correlation between the recalculated metric with that from the original sample). As shown in Figure 4 the centrality metrics appear highly stable. In our analysis, the stability coefficients were 0.28 for betweenness, 0.44 for closeness, and 0.60 for strength, each meeting the 0.25 criteria suggested by Epskamp et al. (2018), indicating high reliability.

**FIGURE 5:**
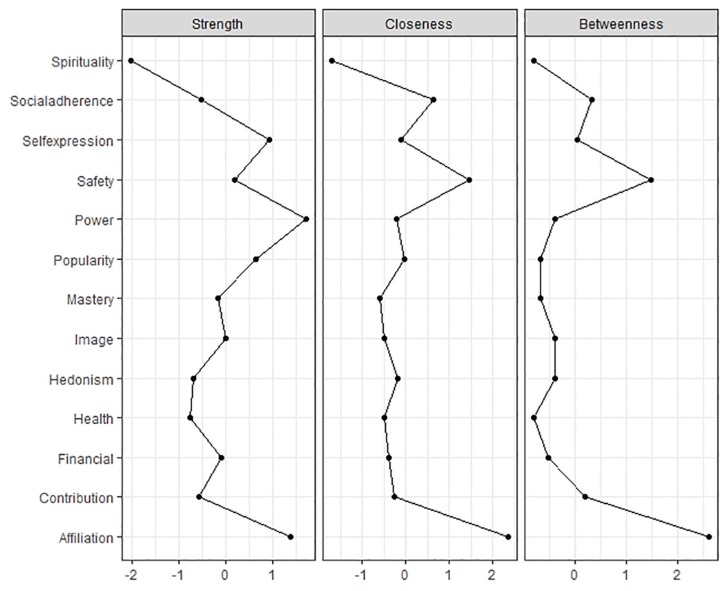
Strength centrality, closeness centrality, and betweenness centrality for each of the 13 aspirations in study 2.

Strength centrality measures how strongly a node is connected to other nodes in the network, betweenness centrality represents the number of times a node falls on the shortest path between two other nodes, and closeness centrality is the average distance from a node to all other nodes in the network (Bell and O’Driscoll, 2018; Epskamp et al., 2018). We examined these indices for each aspiration and included the results in Figure 5. As represented by the strength centrality indices, the aspirations most strongly connected to other aspirations in the network include power, affiliation, self-expression, and popularity, with the least influential node being spirituality. Spirituality is also the least closely connected to all other aspirations (closeness centrality) and plays the smallest role in terms of mediating links between other variables (betweenness). Affiliation and safety have the highest closeness and betweenness centrality, with the other aspirations contributing to a similar degree on these latter metrics.

#### Network Sub-Communities

**FIGURE 6:**
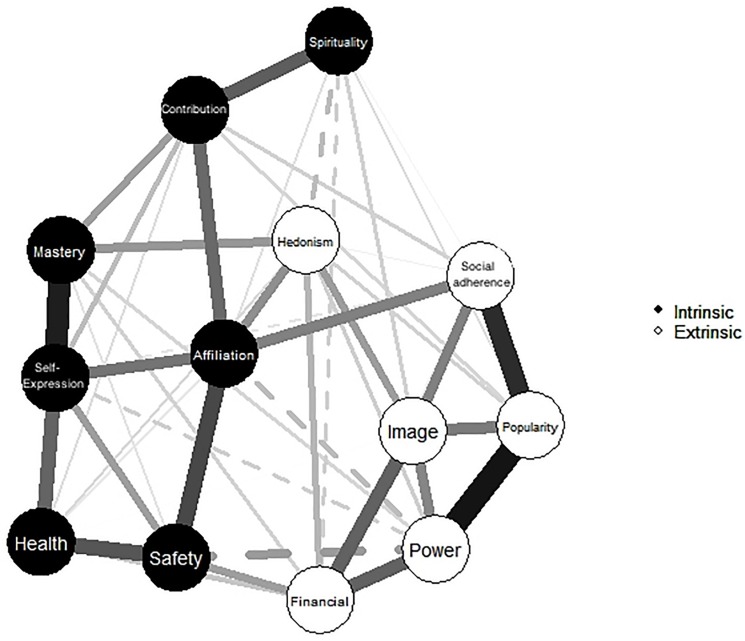
The intrinsic (black nodes) and extrinsic (white nodes) aspiration network sub-communities identified using the spinglass algorithm in study 2.

While the network structure shown in Figure 3 appeared to show the intrinsic/extrinsic distinction, for clarity we also used the spinglass algorithm to identify sub-communities, or clusters, of aspirations within the broader network. Spinglass identifies sub-communities, or clusters, of nodes with many edges within, and few edges between clusters (Bell and O’Driscoll, 2018). Please see Figure 6. The results of this analysis clearly provide support for the intrinsic/extrinsic distinction, classifying health, community contribution, affiliation, self-expression, mastery/skill, safety, and spirituality as intrinsic and clustering popularity, image, financial success, power, social adherence, and hedonism as extrinsic. Interestingly, there remain positive links between some of the intrinsic and extrinsic aspirations. For example, links between safety and financial success, affiliation and social adherence, and hedonism with mastery and image are all positive.

### Relations to Well-Being

The zero-order correlations between various goals and well-being and ill-being indicators in both samples is found in Table 2. As can be seen, most goals had positive zero-order correlations with well-being indicators and negative correlations with ill-being indicators. The mean level of overall aspiring is often positively correlated with well-being and negatively related with ill-being (see e.g., Kasser and Ryan, 1996). Concordantly, in our study, mean aspiring (calculated by averaging all 13 aspirations) was positively correlated with positive affect (0.402 in sample 1, 0.399 in sample 2), life satisfaction (0.308/0.317), vitality (0.449/0.393), meaning (0.392/0.360), and negatively correlated with negative affect (-0.285/-0.162), and stress in sample 2 (-0.198) (all *p*’s < 0.01). To control for this response tendency, and to examine how the relative importance of each individual goal is related to well-being, two strategies have been used in the literature. The first strategy uses regression analysis wherein the importance of the goal of interest and aggregate goal importance are simultaneously regressed on the well-being outcome (e.g., Kasser and Ryan, 1993, 1996). The other strategy involves mean-centering each goal to the participant’s mean rating of overall goal importance, by subtracting the mean across all aspirations from each aspiration (e.g., Kasser and Ryan, 2001). The aim of both methods is to reveal how much participants prioritize each goal relative to other goals, and how that priority is related to well-being. For comprehensiveness, we present both analyses separately. The direct comparison of the two methods using same data could inform future researchers facing the choice of how to account for response tendency in goal research.

**TABLE 2:** The zero-order correlations between study variables in samples 1 and 2.

	**1**	**2**	**3**	**4**	**5**	**6**	**7**	**8**	**9**	**10**	**11**	**12**	**13**	**14**	**15**	**16**	**17**	**18**	**19**	**20**
1. Positive affect		0.711^∗∗^	0.711^∗∗^	0.728^∗∗^	–0.709^∗∗^	–0.625^∗∗^	–0.658^∗∗^	0.377^∗∗^	0.298^∗∗^	0.388^∗∗^	0.422^∗∗^	0.326^∗∗^	0.257^∗∗^	–0.008	0.230^∗∗^	0.072	0.150^∗^	0.124	0.294^∗∗^	0.331^∗∗^
2. Life satisfaction	0.747^∗∗^		0.612^∗∗^	0.646^∗∗^	–0.555^∗∗^	–0.496^∗∗^	–0.599^∗∗^	0.187^∗∗^	0.131	0.365^∗∗^	0.322^∗∗^	0.206^∗∗^	0.195^∗∗^	0.016	0.194^∗∗^	0.061	0.129	0.119	0.213^∗∗^	0.301^∗∗^
3. Vitality	0.753^∗∗^	0.795^∗∗^		0.773^∗∗^	–0.624^∗∗^	–0.471^∗∗^	–0.583^∗∗^	0.332^∗∗^	0.344^∗∗^	0.368^∗∗^	0.391^∗∗^	0.355^∗∗^	0.195^∗∗^	0.191^∗∗^	0.208^∗∗^	0.151^∗^	0.239^∗∗^	0.217^∗∗^	0.331^∗∗^	0.285^∗∗^
4. Meaning	0.686^∗∗^	0.797^∗∗^	0.783^∗∗^		–0.632^∗∗^	–0.559^∗∗^	–0.637^∗∗^	0.319^∗∗^	0.316^∗∗^	0.393^∗∗^	0.406^∗∗^	0.279^∗∗^	0.242^∗∗^	0.069	0.131	0.102	0.14	0.165^∗^	0.237^∗∗^	0.364^∗∗^
5. Negative Affect	–0.621^∗∗^	–0.625^∗∗^	–0.624^∗∗^	–0.588^∗∗^		0.692^∗∗^	0.689^∗∗^	–0.289^∗∗^	–0.231^∗∗^	−0.266^∗∗^−	0.302^∗∗^	–0.206^∗∗^	0.207^∗∗^	–0.044	−0.151^∗^	–0.089	–0.108	–0.044	0.209^∗∗^	–0.194^∗∗^
6. Anxiety	–0.506^∗∗^	–0.518^∗∗^	–0.557^∗∗^	–0.531^∗∗^	0.765^∗∗^		0.709^∗∗^	–0.206^∗∗^	−0.148^∗^	−0.188^∗∗^−	0.264^∗∗^	−0.154^∗^	–0.126	0.106	–0.047	0.057	0.026	0.094	–0.103	–0.109
7. Stress	–0.650^∗∗^	–0.649^∗∗^	–0.676^∗∗^	–0.587^∗∗^	0.751^∗∗^	0.717^∗∗^		–0.219^∗∗^	−0.180^∗^	−0.233^∗∗^−	0.236^∗∗^	−0.168^∗^	0.201^∗∗^	0.135	0.03	0.096	0.118	0.156^∗^	–0.074	–0.06
8. Self-expression	0.325^∗∗^	0.220^∗∗^	292^∗∗^	0.282^∗∗^	–0.169^∗∗^	−0.112^∗^	–0.230^∗∗^		0.594^∗∗^	0.619^∗∗^	0.522^∗∗^	0.563^∗∗^	0.566^∗∗^	–0.091	0.217^∗∗^	0.314^∗∗^	0.148^∗^	0.077	0.386^∗∗^	0.157^∗^
9. Mastery	0.352^∗∗^	0.283^∗∗^	0.415^∗∗^	0.312^∗∗^	–0.173^∗∗^	−0.138^∗^	–0.255^∗∗^	0.739^∗∗^		0.530^∗∗^	0.552^∗∗^	0.569^∗∗^	0.407^∗∗^	0.087	0.180^∗^	0.322^∗∗^	0.212^∗∗^	0.13	0.342^∗∗^	0.138
10. Affiliation	0.369^∗∗^	0.336^∗∗^	0.308^∗∗^	0.364^∗∗^	–0.145^∗∗^	–0.057	–0.183^∗∗^	0.690^∗∗^	0.586^∗∗^		0.501^∗∗^	0.550^∗∗^	0.632^∗∗^	–0.121	0.298^∗∗^	0.201^∗∗^	0.145^∗^	0.122	0.370^∗∗^	0.229^∗∗^
11. Contribution	0.359^∗∗^	0.271^∗∗^	0.338^∗∗^	0.365^∗∗^	−0.134^∗^	–0.068	−0.114^∗^	0.567^∗∗^	0.560^∗∗^	0.624^∗∗^		0.457^∗∗^	0.357^∗∗^	–0.008	0.329^∗∗^	0.162^∗^	0.214^∗∗^	0.138	0.201^∗∗^	0.377^∗∗^
12. Health	0.359^∗∗^	0.211^∗∗^	0.307^∗∗^	0.250^∗∗^	–0.177^∗∗^	−0.111^∗^	–0.256^∗∗^	0.697^∗∗^	0.628^∗∗^	0.599^∗∗^	0.495^∗∗^		0.563^∗∗^	–0.044	0.281^∗∗^	0.378^∗∗^	0.121	0.197^∗∗^	0.325^∗∗^	0.124
13. Safety	0.266^∗∗^	0.180^∗∗^	0.130^∗^	0.199^∗∗^	–0.102	–0.004	–0.192^∗∗^	0.664^∗∗^	0.560^∗∗^	0.687^∗∗^	0.461^∗∗^	0.664^∗∗^		−0.178^∗^	0.208^∗∗^	0.304^∗∗^	0.029	0.013	0.197^∗∗^	0.108
14. Power	0.189^∗∗^	0.173^∗∗^	0.257^∗∗^	0.180^∗∗^	−0.126^∗^	−0.125^∗^	–0.097	0.015	0.253^∗∗^	0.061	0.169^∗∗^	0.095	–0.05		0.461^∗∗^	0.554^∗∗^	0.697^∗∗^	0.650^∗∗^	0.422^∗∗^	0.186^∗∗^
15. Social adherence	0.250^∗∗^	0.261^∗∗^	0.273^∗∗^	0.247^∗∗^	–0.087	–0.061	−0.133^∗^	0.246^∗∗^	0.345^∗∗^	0.487^∗∗^	0.437^∗∗^	0.293^∗∗^	0.318^∗∗^	0.493^∗∗^		0.538^∗∗^	0.746^∗∗^	0.684^∗∗^	0.418^∗∗^	0.339^∗∗^
16. Financial success	0.09	0.036	0.117^∗^	0.063	0.013	0.046	–0.049	0.313^∗∗^	0.450^∗∗^	0.332^∗∗^	0.272^∗∗^	0.426^∗∗^	0.392^∗∗^	0.571^∗∗^	0.507^∗∗^		0.605^∗∗^	0.609^∗∗^	0.536^∗∗^	0.182^∗^
17. Popularity	0.218^∗∗^	0.172^∗∗^	0.227^∗∗^	0.172^∗∗^	–0.066	–0.036	–0.077	0.177^∗∗^	0.320^∗∗^	0.274^∗∗^	0.379^∗∗^	0.210^∗∗^	0.154^∗∗^	0.736^∗∗^	0.708^∗∗^	0.550^∗∗^		0.741^∗∗^	0.600^∗∗^	0.257^∗∗^
18. Image	0.187^∗∗^	0.152^∗∗^	0.245^∗∗^	0.169^∗∗^	–0.038	–0.008	–0.038	0.218^∗∗^	0.342^∗∗^	0.310^∗∗^	0.300^∗∗^	0.322^∗∗^	0.198^∗∗^	0.656^∗∗^	0.643^∗∗^	0.645^∗∗^	0.710^∗∗^		0.500^∗∗^	0.285^∗∗^
19. Hedonism	0.307^∗∗^	0.213^∗∗^	0.277^∗∗^	0.153^∗∗^	−0.122^∗^	–0.075	–0.144^∗∗^	0.441^∗∗^	0.523^∗∗^	0.514^∗∗^	0.368^∗∗^	0.446^∗∗^	0.378^∗∗^	0.418^∗∗^	0.478^∗∗^	0.527^∗∗^	0.492^∗∗^	0.541^∗∗^		0.029
20. Spirituality	0.207^∗∗^	0.200^∗∗^	0.199^∗∗^	0.301^∗∗^	–0.094	−0.120^∗^	–0.045	0.199^∗∗^	0.196^∗∗^	0.262^∗∗^	0.401^∗∗^	0.190^∗∗^	0.174^∗∗^	0.199^∗∗^	0.242^∗∗^	0.052	0.250^∗∗^	0.210^∗∗^	0.034	

Study 1 correlations above the diagonal, study 2 correlations below the diagonal. ^∗^Correlation is significant at the 0.5 level (2-tailed).*^∗^*Correlation is significant at the 0.01 level (2-tailed).*

#### Relations to Well-Being Using Regression Analysis

To examine the relations of intrinsic and extrinsic goals on well-being and ill-being, we followed previous researchers (e.g., Romero et al., 2012) by regressing overall extrinsic and intrinsic goal importance on various well-being indicators. More specifically, we first generated a summary score of intrinsic goal importance by averaging together all six intrinsic goal scores, and similarly averaged together the six extrinsic goal scores. Extrinsic and intrinsic goal importance correlated at 0.266/0.449. Then we conducted regression analyses using each well-being/ill-being indicator in turn as the dependent variable and with the summary scores for intrinsic goal importance and extrinsic goal importance entered simultaneously as independent variables to control for each other’s influence. Given that both intrinsic and extrinsic aspirations were in general positively correlated with well-being indicators, and negatively correlated with ill-being indicators, it was interesting to see whether one of them had more robust relations with well-being. The results of the regression analysis thus display whether intrinsic aspirations are connected to well-being even when controlling for the influence of extrinsic aspirations, and whether extrinsic aspirations are connected to well-being when controlling for intrinsic aspirations.

Results are displayed in Table 3 and show that, as expected, aspiring for intrinsic goals was positively associated with all four well-being indicators (positive affect, life satisfaction, vitality, and meaning in life) in both samples, and negatively associated with all three ill-being indicators (negative affect, anxiety, and stress), except for anxiety in sample 2. Extrinsic aspirations in turn, mostly showed no relation to well-being or ill-being indicators when controlling for the effect of intrinsic aspirations. The only significant relations were the positive relationship with vitality, and a positive relationship with stress in sample 1.

**TABLE 3:** The relations of intrinsic and extrinsic aspirations on various indicators of well-being and ill-being, when controlling for each other’s influence.

	**Positive affect**		**Life satisfaction**		**Vitality**		**Meaning**		**Negative affect**		**Anxiety**		**Stress**	
							
	**β**	***p***	**β**	***p***	**β**	***p***	**β**	***p***	**β**	***p***	**β**	***p***	**β**	***p***
Intrinsic aspirations	0.428/ 0.374	<0.001/ <0.001	0.284/ 0.268	<0.001/ <0.001	0.381/ 0.296	<0.001/ <001	0.403/ 0.342	<0.001/ <0.001	−0.308/ −0.176	<0.001/ 0.003	−0.260/ −0.092	<0.001/ 0.131	−0.312/ −0.243	<0.001/ <0.001
Extrinsic aspirations	0.064/ 0.091	0.338/ 0.102	0.076/ 0.091	0.284/ 0.115	0.175/ 0.158	0.009/ 0.005	0.067/ 0.052	0.326/ 0.356	−0.050/ −0.012	0.477/ 0.847	0.097/ −0.016	0.181/ 0.794	0.179/ −0.003	0.012/ 0.955

Going beyond the relation between overall well-being and overall intrinsic and extrinsic aspirations, we wanted to look how individual aspirations related to these varied indicators of well- and ill-being. As we were interested in the relative importance of a single aspiration as compared to the importance of the rest of aspirations, we generated for each aspiration a control variable by averaging all the 12 other aspirations except for the one aspiration under scrutiny, which was entered into regression analysis at step 1. The full results including standardized beta coefficients and their statistical significance are displayed in Table 4. To lower the risk of false positives given the number of relations examined, we discuss here only those results that were consistent across both samples (significant or at least marginally significant in both). Of the various intrinsic aspirations, *self-expression* had positive relations with positive affect and meaning as well as total well-being, while having negative relations with negative affect, stress, and total ill-being. *Mastery* had positive relations with positive affect, vitality, and meaning, and negative relations with stress and total ill-being. *Affiliation* was positively related to positive affect, life satisfaction, meaning and total well-being, while *community contribution* had positive associations with positive affect, life satisfaction, vitality, meaning, and total well-being. The only consistent relation for *safety* was a negative association with stress, supporting its role as a deficit need (see Ryan and Deci, 2017). *Health* was positively associated with positive affect, vitality, and total well-being, and negatively associated with stress and total ill-being. Interestingly, *spirituality*, which was not classified as intrinsic, had positive relations with positive affect, life satisfaction, vitality, meaning and total well-being.

**TABLE 4:** The relations of various life goals on the well-being and ill-being indicators, when using regression analysis to control for the overall goal striving.

	**Total well-being**		**Positive affect**		**Life satisfaction**		**Vitality**		**Meaning**		**Total ill-being**		**Negative affect**		**Anxiety**		**Stress**	
									
			**β**	***p***	**β**	***p***	**β**	***p***	**β**	***p***	**β**	***p***	**β**	***p***	**β**	***p***	**β**	***p***
Self-expression	0.169/ 0.111	0.025/ 0.072	0.246/ 0.145	0.001/ 0.019	0.045/ 0.054	0.574/0.397	0.148/ 0.097	0.047/ 0.117	0.171/ 0.112	0.026/ 0.075	−0.243/ −0.133	003/ 0.045	−0.205/ −0.118	0.010/ 0.075	−0.205/ −0.081	0.013/ 0.226	−0.251/ −0.183	0.002/ 0.005
Mastery	0.114/ 0.198	0.130/ 0.003	0.130/ 0.164	0.090/ 0.014	−0.039/ 0.137	0.621/ 0.049	0.162/ 0.294	0.031/ <0.001	0.164/ 0.137	0.034/ 0.045	−0.159/ −0.174	0.053/ 0.016	−0.121/ −0.124	0.132/ 0.084	−0.123/ −0.131	0.141/ 0.071	−0.198/ −0.237	0.017/ 0.001
Affiliation	0.295/ 0.217	<0.001/ 0.001	0.261/ 0.201	0.001/ 0.002	0.296/ 0.242	<0.001/ <0.001	0.197/ 0.093	0.009/ 0.163	0.274/ 0.240	<0.001/ <0.001	−0.226/ −0.045	0.006/ 0.525	−0.171/ −0.072	0.033/ 0.313	−0.180/ 0.022	0.030/ 0.754	−0.273/ −0.098	0.001/ 0.164
Contribution	0.309/ 0.202	<0.001/ 0.002	0.313/ 0.193	<0.001/ 0.003	0.239/ 0.129	0.002/ 0.051	0.238/ 0.161	0.001/ 0.012	0.298/ 0.247	<0.001/ <0.001	−0.294/ −0.016	<0.001/ 0.817	−0.226/ −0.057	0.004/ 0.407	−0.287/ −0.004	<0.001/ 0.951	−0.274/ 0.019	0.001/ 0.781
Safety	0.103/ −0.008	0.144/ 0.895	0.125/ 0.075	0.080/ 0.209	0.093/ 0.012	0.209/ 0.846	0.027/ −0.123	0.700/ 0.038	0.111/ 0.006	0.122/ 0.920	−0.150/ −0.011	0.050/ 0.860	−0.119/ −0.020	0.111/ 0.750	−0.096/ 0.078	0.212/ 0.225	−0.206/ −0.126	0.007/ 0.047
Health	0.149/ 0.105	0.047/ 0.094	0.174/ 0.197	0.023/ 0.002	0.071/ 0.032	0.375/ 0.620	0.182/ 0.115	0.015/ 0.066	0.114/ 0.054	0.139/ 0.400	0.146/ −0.150	0.075/ 0.025	−0.088/ −0.131	0.272/ 0.051	−0.133/ −0.080	0.109/ 0.241	−0.180/ −0.227	0.030/ 0.001
Power	−0.123/ 0.038	0.077/ 0.497	−0.208/ −0.001	0.003/ 0.993	−0.132/ 0.032	0.074/ 0.586	0.017/ 0.098	0.805/ 0.086	−0.107/ 0.013	0.133/ 0.825	0.180/ −0.071	0.018/ 0.245	0.085/ −0.068	0.255/ 0.265	0.192/ −0.103	0.012/ 0.092	0.210/ −0.004	0.006/ 0.941
Social adherence	−0.169/ 0.027	0.051/ 0.683	−0.087/ −0.035	0.327/ 0.600	−0.030/ 0.096	0.744/ 0.164	−0.197/ 0.018	0.022/ 0.792	−0.275/ 0.010	0.002/ 0.880	0.116/ 0.023	0.223/ 0.748	0.086/ 0.043	0.358/ 0.549	0.071/ 0.018	0.463/ 0.805	0.174/ 0.001	0.072/ 0.994
Popularity	−0.208/ −0.088	0.014/ 0.175	−0.228/ −0.079	0.008/ 0.226	−0.147/ −0.064	0.104/ 0.342	−0.115/ −0.054	0.176/ 0.406	−0.237/ −0.119	0.006/ 0.073	0.253/ 0.083	0.007/ 0.241	0.159/ 0.074	0.082/ 0.293	0.207/ 0.061	0.028/ 0.391	0.338/ 0.098	<0.001/ 0.163
Financial	−0.346/ −0.299	<0.001/ <0.001	−0.370/ −0.281	<0.001/ <0.001	−0.273/ −0.285	0.002/ <0.001	−0.279/ −0.226	<0.001/ <0.001	−0.302/ −0.286	<0.001/ <0.001	0.274/ 0.197	0.003/ 0.004	0.190/ 0.201	0.036/ 0.003	0.261/ 0.195	0.005/ 0.004	0.288/ 0.132	0.002/ 0.052
Hedonism	0.075/ 0.004	0.350/ 0.950	0.096/ 0.098	0.236/ 0.129	0.055/ 0.077	0.517/ 0.729	0.112/ 0.051	0.159/ 0.433	0.013/ −0.134	0.876/ 0.039	−0.062/ −0.029	0.475/ 0.672	−0.070/ −0.034	0.409/ 0.622	−0.053/ −0.016	0.549/ 0.815	−0.042/ 0.043	0.637/ 0.628
Image	−0.157/ −0.115	0.051/ 0.079	−0.211/ −0.147	0.010/ 0.025	−0.119/ −0.109	0.166/ 0.110	−0.100/ −0.027	0.215/ 0.678	−0.130/ −0.131	0.115/ 0.050	0.320/ 0.152	<0.001/ 0.032	0.224/ 0.131	0.009/ 0.065	0.290/ 0.116	0.001/ 0.104	0.358/ 0.178	<0.001/ 0.011
Spirituality	0.264/ 0.153	<0.001/ 0.003	0.237/ 0.102	0.001/ 0.051	0.238/ 0.122	0.001/ 0.024	0.164/ 0.094	0.016/ 0.072	0.279/ 0.224	<0.001/ <0.001	−0.093/ −0.059	0.212/ 0.293	−0.120/ −0.053	0.102/ 0.347	−0.083/ −0.103	0.272/ 0.069	−0.039/ 0.016	0.605/ 0.777

Of the extrinsic goals, *popularity* was negatively associated with meaning. *Financial success* had negative associations with all four well-being indicators and total well-being, and positive associations with all three ill-being indicators and total ill-being. *Image* was negatively associated with positive affect and total well-being, and positively associated with negative affect, stress, and total ill-being. *Power, social adherence*, and *hedonism* didn’t have any consistent relations with any well-being or ill-being indicator.

#### Relations to Well-Being Using Mean-Centering

For this analysis, we mean-centered each goal importance to the participant’s mean rating of goal importance (calculated by averaging the importance of all 13 goals for each participant).

Using these mean-centered goal importance scores, we then examined their relations to the various well-being and ill-being indicators. Results are displayed in Table 5 and show that, as expected, aspiring for intrinsic goals was positively associated with meaning and positive affect (marginally significantly in sample 2), and negatively associated with stress (marginally significantly in sample 2). Aspiring for extrinsic goals, in turn, was negatively associated with meaning and positive affect (marginally significantly in sample 2), and positively associated with stress (marginally significantly in sample 2).

**TABLE 5:** The link between mean-centered global and specific goals and well-being indicators.

	**Total ill-being**		**Positive affect**		**Life satisfaction**		**Vitality**		**Meaning**		**Total ill-being**		**Negative Affect**		**Anxiety**		**Stress**	
									
	**β**	***p***	**β**	***p***	**β**	***p***	**β**	***p***	**β**	***p***	**β**	***p***	**β**	***p***	**β**	***p***	**β**	***p***
Total intrinsic	0.12/ 0.08	0.104/ 0.136	0.15/ 0.10	0.031/ 0.058	0.08/ 0.06	0.278/ 0.290	0.05/ 0.03	0.473/ 0.608	0.14/ 0.11	0.049/ 0.037	−0.22/ −0.07	0.002/ 0.171	−0.11/ −0.07	0.131/ 0.213	−0.19/ −0.03	0.007/ 0.592	−0.28/ −0.10	<0.001/ 0.063
Total extrinsic	−0.12/ −0.08	0.104/ 0.136	−0.15/ −0.10	0.031/ 0.058	−0.08/ −0.06	0.278/ 0.290	−0.05/ −0.03	0.473/ 0.608	−0.14/ −0.11	0.049/ 0.037	0.22/ 0.07	0.002/ 0.171	0.11/ 0.07	0.131/ 0.213	0.19/ 0.03	0.007/ 0.592	0.28/ 0.10	<0.001/ 0.063
Self-Expression	0.02/ −0.04	0.818/ 0.473	0.10/ −0.01	0.165/ 0.868	−0.05/ −0.06	0.519/ 0.311	0.00/ −0.04	0.964/ 0.412	0.04/ −0.02	0.596/ 0.672	−0.16/ −0.06	0.03/ 0.28	−0.10/ −0.04	0.179/ 0.429	−0.14/ −0.03	0.048/ 0.562	−0.19/ −0.08	0.007/ 0.127
Mastery	−0.03/ 0.10	0.660/ 0.070	−0.01/ 0.07	0.933/ 0.184	−0.12/ 0.06	0.088/ 0.255	0.01/ 0.17	0.923/ 0.002	0.02/ 0.06	0.730/ 0.298	−0.09/ −0.12	0.23/ 0.03	−0.02/ −0.07	0.780/ 0.184	−0.07/ −0.08	0.339/ 0.120	−0.14/ −0.15	0.046/ 0.005
Affiliation	0.14/ 0.15	0.043/ 0.006	0.12/ 0.14	0.084/ 0.012	0.18/ 0.17	0.013/ 0.002	0.06/ 0.05	0.425/ 0.321	0.14/ 0.17	0.055/ 0.002	−0.15/ −0.04	0.030/ 0.506	−0.08/ −0.05	0.289/ 0.379	−0.12/ 0.02	0.083/ 0.696	−0.21/ −0.07	0.003/ 0.222
Contribution	0.19/ 0.16	0.008/ 0.003	0.20/ 0.15	0.004/ 0.004	0.15/ 0.10	0.031/ 0.056	0.13/ 0.13	0.074/ 0.017	0.19/ 0.20	0.007/ <0.001	−0.22/ −0.01	0.002/ 0.812	−0.15/ −0.05	0.040/ 0.397	−0.23/ −0.00	0.001/ 0.937	−0.22/ 0.01	0.002/ 0.812
Safety	−0.09/ −0.14	0.215/ 0.007	−0.05/ −0.07	0.466/ 0.186	−0.04/ −0.10	0.556/ 0.078	−0.16/ −0.23	0.027/ <0.001	−0.06/ −0.11	0.396/ 0.034	−0.07/ −0.03	0.356/ 0.549	0.01/ 0.04	0.886/ 0.492	−0.04/ 0.10	0.615/ 0.069	−0.15/ −0.04	0.038/ 0.496
Health	0.04/ −0.03	0.599/ 0.600	0.07/ 0.05	0.332/ 0.346	0.00/ −0.06	0.990/ 0.295	0.07/ −0.01	0.354/ 0.835	0.02/ −0.05	0.787/ 0.346	−0.09/ −0.08	0.199/ 0.120	−0.02/ −0.06	0.794/ 0.261	−0.09/ −0.04	0.208/ 0.515	−0.14/ −0.13	0.052/ 0.019
Power	−0.18/ −0.02	0.012/ 0.723	−0.26/ −0.06	<0.001/ 0.297	−0.17/ −0.02	0.015/ 0.766	−0.06/ 0.03	0.411/ 0.563	−0.16/ −0.04	0.021/ 0.469	0.20/ −0.03	0.005/ 0.575	0.13/ −0.04	0.077/ 0.491	0.20/ −0.08	0.005/ 0.156	0.21/ 0.02	0.003/ 0.660
Social Adherence	−0.04/ 0.05	0.557/ 0.313	0.01/ 0.00	0.850/ 0.928	0.04/ 0.10	0.614/ 0.078	−0.06/ 0.04	0.442/ 0.424	−0.12/ 0.04	0.089/ 0.515	0.05/ 0.00	0.446/ 0.959	0.01/ 0.02	0.918/ 0.722	0.03/ 0.01	0.698/ 0.925	0.11/ −0.01	0.131/ 0.784
Popularity	−0.08/ −0.05	0.242/ 0.386	−0.11/ −0.04	0.137/ 0.460	−0.06/ −0.03	0.388/ 0.542	−0.02/ −0.02	0.809/ 0.687	−0.11/ −0.07	0.110/ 0.184	0.17/ 0.06	0.020/ 0.297	0.07/ 0.05	0.305/ 0.376	0.13/ 0.04	0.062/ 0.451	0.23/ 0.06	0.001/ 0.238
Money	−0.26/ −0.27	<0.001/ <0.001	−0.29/ −0.26	<0.001/ <0.001	−0.21/ −0.26	0.003/ <0.001	−0.22/ −0.22	0.002/ <0.001	−0.23/ −0.26	0.001/ <0.001	0.21/ 0.17	0.003/ 0.002	0.15/ 0.17	0.038/ 0.001	0.20/ 0.16	0.005/ 0.002	0.22/ 0.12	0.002/ 0.023
Hedonism	0.06/ −0.01	0.409/ 0.858	0.08/ 0.07	0.270/ 0.201	0.05/ 0.01	0.530/ 0.813	0.09/ 0.03	0.201/ 0.543	0.01/ −0.11	0.876/ 0.042	−0.05/ −0.02	0.483/ 0.694	−0.06/ −0.02	0.422/ 0.660	−0.04/ −0.01	0.548/ 0.839	−0.03/ −0.02	0.636/ 0.675
Image	−0.08/ −0.08	0.246/ 0.164	−0.13/ −0.10	0.062/ 0.058	−0.07/ −0.08	0.339/ 0.163	−0.04/ −0.01	0.556/ 0.812	−0.07/ −0.09	0.323/ 0.092	0.24/ 0.12	0.001/ 0.034	0.15/ 0.10	0.032/ 0.079	0.22/ 0.09	0.002/ 0.115	0.27/ 0.13	<0.001/ 0.016
Spirituality	0.20/ 0.09	0.004/ 0.104	0.18/ 0.04	0.011/ 0.507	0.19/ 0.07	0.007/ 0.207	0.11/ 0.03	0.134/ 0.587	0.22/ 0.16	0.002/ 0.003	−0.07/ −0.02	0.360/ 0.722	−0.08/ −0.03	0.248/ 0.636	−0.07/ −0.08	0.357/ 0.125	−0.03/ −0.05	0.693/ 0.391

*The general intrinsic and extrinsic life goals have been mean centered by subtracting the mean across 12 of the 13 aspirations, excluding spirituality, because spirituality docs not feature in the mean of intrinsic or extrinsic aspirations. Otherwise, the mean across all 13 life goals has been subtracted from the mean for each individual aspiration. The mean-centering procedure accounts for response tendency and situates each life goal relative to other life goals.*

We also examined the relations of individual aspirations to various well-being and ill-being indicators. These results are also displayed in Table 5. Given the number of relations examined, we again discuss here only those results that were consistent across both samples (significant or at least marginally significant in both). Using the mean-centered priorities for each individual goal, of various intrinsic goals, *mastery* had significant negative relations with stress, *affiliation* had significant positive relations with total well-being, SWLS, positive affect, and meaning (the latter two were marginally significant in sample 1), *contribution* had positive relations with total well-being, meaning, positive affect, SWLS and vitality (the latter two were marginally significant in sample 2), *safety* had significant negative relations with vitality, and *health* had significant negative relations with stress (marginally significant in sample 1). *Self-expression* didn’t have any consistent relations to well-being indicators, although it was negatively related to anxiety and stress in sample 1. Of the various extrinsic goals, *financial success* was positively related to total ill-being, negative affect, stress, and anxiety, and negatively related to total well-being, positive affect, SWLS, meaning, and vitality, and *image* was positively related to total ill-being, negative affect (marginally significantly in sample 2), and stress. *Power, social adherence, popularity*, and *hedonism* didn’t have any consistent significant relations with well-being or ill-being indicators. *Spirituality* was significantly and positively related to meaning.

## Discussion

Using a combination of traditional and novel analytic strategies, we sought to extend the map of intrinsic and extrinsic aspirations by including aspirations for self-expression, mastery, power, and social adherence as additional life goals, and by assessing the links between these candidate aspirations and established intrinsic and extrinsic goals, and with indicators of psychological wellness. We expected self-expression and mastery to group with the intrinsic aspirations, and power and social adherence to group with the extrinsic aspirations through the use of factor analysis, multidimensional scaling, and the novel application of EBICglasso network analysis. We also expected positive links between wellness and the candidate intrinsic aspirations and negative or neutral links with the candidate extrinsic aspirations. The results largely confirmed our hypotheses, as outlined below. These results also suggest possibilities for researchers to test additional and novel life aspirations using the mapping strategies illustrated herein.

### Structure of Aspirations and Evaluation of Candidate Life Goals

The structure of aspirations derived using factor analysis, multidimensional scaling, and circumplex configuration was consistent with past results demonstrating the intrinsic and extrinsic distinction (Kasser and Ryan, 1996; Grouzet et al., 2005). In addition, and consistent with hypotheses, these analyses showed that self-expression and mastery grouped clearly with other intrinsic goals in both samples, while power and social adherence clustered with other extrinsic goals. This provides evidence for concluding that self-expression and mastery should be seen as intrinsic goals, while power and social adherence should be seen as extrinsic goals.

A methodological contribution of the present article was to complement these traditional analyses through the novel use of EBICglasso network analysis and the spinglass algorithm. This approach allowed us to visualize a model-free network of aspirations derived from partial correlations between each pair of aspirations, controlling for all other goals in the network, and to identify sub-communities of aspirations comprising the network. The added benefit of network analysis is its ability to visualize links between nodes in the network, which are not observed using multidimensional scaling or in the circumplex structure. Strong positive links were found between the widely accepted extrinsic aspirations for financial success, popularity, and image on the one hand, and strong positive links were also found between the intrinsic aspirations for community contribution, affiliation, and health on the other (Kasser and Ryan, 2001). In addition, self-expression and mastery were enmeshed with the other intrinsic aspirations, linking strongly to most other nodes in this sub-community. Conformity and power also linked strongly to other nodes in the extrinsic sub-community. Furthermore, aspirations for power linked negatively with the intrinsic aspirations for affiliation, self-expression, and safety. These links are intuitive and demonstrate the utility of including power as an extrinsic aspiration.

Notably, there were some links between several intrinsic and extrinsic nodes, which may help explain why intrinsic and extrinsic aspirations are often positively correlated in zero-order correlations (Bradshaw et al., 2018b). Often these links were sensible, such as the correlation between safety (intrinsic) and financial success (extrinsic). For some, money may feel like a path to safety. In addition, the link between social adherence (extrinsic) and affiliation (intrinsic) suggests that for some, a desire for relatedness may manifest in the want to “fit in.” Taken together, the network analysis results supported both the previously reported distinction of aspirations into intrinsic and extrinsic, and our hypotheses about self-expression and mastery being positioned in the intrinsic sub-community of the network, and power and conformity being situated in the extrinsic sub-community.

The network analysis also provided information about the network centrality (or lack thereof) of specific aspirations. In this regard, spirituality demonstrated the least centrality, or influence, on each of the three centrality metrics. The low strength, centrality, and betweenness scores for spirituality suggest that it is the least strongly connected, the least widely connected, and the least involved in mediating links between other aspirations. The intrinsic/extrinsic quality of spirituality aspirations has been questioned previously (Grouzet et al., 2005), in part because much SDT research suggests that different motives can underlie spiritual and religious pursuits (e.g., Ryan et al., 1993). We therefore suggest omitting spirituality in studies that concentrate on the intrinsic and extrinsic distinction, although assessing spirituality as a life goal should otherwise remain an area of interest (e.g., Martos et al., 2011; Brambilla et al., 2015). Of the remaining aspirations, the most influential include affiliation, safety, popularity, image, and the newly included aspirations for power, social adherence, mastery, and self-expression.

Each of the four distinct statistical techniques used in this study placed our four candidate aspirations in their hypothesized domains. Additionally, measures of centrality indicated they also play an influential role in the structure of the network of aspirations. Taken together, these consistent results provide a reliable rationale for including the candidate goals in the study of intrinsic and extrinsic aspirations. Moreover, the replication of the intrinsic/extrinsic domains using a novel analytic approach demonstrates the robustness of the distinction, and sheds new light on the links between the aspirations and the influence of particular life goals within the network of aspirations. Indeed, Bradshaw et al. (2018b) found that the configuration of aspirations explains well-being outcomes over and above individual aspirations, so there certainly appears to be utility in considering aspirations as a network as well as individually or dichotomously.

### Candidate Life Goals and Psychological Wellness

To examine the relation of goals to well-being, we utilized two approaches, regression analysis and mean-centering. When comparing the results of these two analyses, they seemed not to contradict each other. However, the mean-centering appeared to be more conservative, resulting in fever statistically significant relations.

When using the regression approach to examine relations to well-being, self-expression had expected positive relations with positive affect, meaning, and total well-being, and negative associations with negative affect, stress, and total ill-being. Mastery had positive relations with positive affect, vitality, and meaning, and negative associations with stress, and total ill-being. When using the mean-centering approach, self-expression didn’t have any consistent significant relations with well-being or ill-being indicators, and mastery had a significant negative association with stress. Both self-expression and mastery thus demonstrated relations that were in the expected direction, although mean-centering found only a few significant relations. Of the other intrinsic goals, affiliation and community contribution also demonstrated consistent and expected positive relations with various well-being indicators using both methods. Safety had negative relations with stress using regression approach, but contrary to predictions had negative association with vitality using mean-centering approach. This result merits further examination, as an intrinsic goal one would have expected the opposite relations. As a potential deficit need (Ryan and Deci, 2017; Martela and Ryan, 2019) safety might not be so salient goal in moments when one feels vital and full of energy. Of the extrinsic goals, power and social adherence did not have consistent relations with either well-being or ill-being indicators, and this was true as regards both analytic approaches, consistent with the idea that extrinsic goals have either a neutral or negative relation with well-being. Of the other extrinsic aspirations, financial success and image were most consistently associated with more ill-being and less well-being, and this was true using both analytic approaches.

Our intent in this study was to focus on the interrelations among aspirations, and their differential associations with various outcomes. For these purposes, our Mturk samples were apt. However, all participants were from the United States, limiting cross-cultural generalizations. Similar models should be tested in multiple cultures, and network analyses may be especially useful in this regard in revealing differential patterns of aspirational strength. An additional limitation is the cross-sectional nature of the study, which limits our ability to make causal inferences concerning the relations between aspirations and outcomes. Especially regarding any new intrinsic and extrinsic goals, it would be important to carry out longitudinal research on their effects, as has been done in some prior research (see Kasser et al., 2014).

Despite these limitations, we believe that this study makes a strong contribution to the literature through the combined use of classical and contemporary statistical approaches to validate the structure of aspirations, and by opening the path to studying self-expression, mastery, social adherence and power as life goals alongside other intrinsic and extrinsic aspirations. Although striving for goals such as power might not be conducive to well-being, striving to express oneself and develop mastery seem akin to other intrinsic goals in their associations, supporting the value of these strivings for wellness. The findings also suggest new strategies for researchers who may wish to examine yet other novel aspirations in relation to the intrinsic and extrinsic goal distinction.

## Data Availability STatement

The datasets generated and analyzed for this study as well as the scripts to replicate the analyses can be found in the OSF: https://osf.io/pt6c9/?view_only=141a1aa535d3454b92ab645c53e60797.

## Ethics Statement

This study was carried out in accordance with the recommendations of the Ethical Review Board of the Aalto University. In accordance with the Declaration of Helsinki, we sought informed consent from all study participants, and they gave their consent anonymously in the online form.

## Author Contributions

FM and RR contributed to the initial conception and design of the study. FM gathered the data, performed the other statistical analyses, and wrote the first draft of the manuscript. EB performed the network analysis and wrote the sections related to network analysis. FM, EB, and RR all contributed to manuscript revision, read and approved the submitted version.

## Conflict of Interest

The authors declare that the research was conducted in the absence of any commercial or financial relationships that could be construed as a potential conflict of interest.

## Footnotes

1 Previous research has examined conformity as the goal of fitting in and blending in with others with items such as “I will be polite and obedient,” and “My desires and tastes will be similar to those of other people” (Grouzet et al., 2005). We see this as somewhat overlapping yet different social goal in that we don’t focus on blending in with others but only on being included. With studying social adherence we thus wanted to focus only on the desire to fit in and not be excluded from the group.

## Appendix

### The aspiration scales for measuring power, social adherence, self-expression, and mastery

**Table:**

**Power**	**Self-expression**
1. I will be powerful.	1. I will be free to do things that really interest me.
2. I will have a high position.	2. I will choose what I do, instead of being pushed along by life.
3. I will be able to command people.	3. My way of living will be in harmony with who I really am.
4. People will obey me.	4. I will have insight into why I do the things I do.

**Social adherence**	**Mastery**

1. I will be accepted by my peers	1. I will be able to do things that I am especially skillful at.
2. I will be approved by people around me.	2. I will set goals and make sure that I achieve them.
3. I will be included in social circles.	3. I will seek difficult tasks that I can master.
4. I will “fit in” with others.	4. I will be efficient.

## References

[B1] Asch S. E. (1956). Studies of independence and conformity: I. A minority of one against a unanimous majority. psychological monographs. *Gen. Appl.* 70 1–70. 10.1037/h0093718

[B2] Bell V. O’Driscoll C. (2018). The network structure of paranoia in the general population. *Soc. Psychiatry Psychiatr. Epidemiol.* 53 737–744. 10.1007/s00127-018-1487-0 29427197 PMC6003969

[B3] Bilsky W. Schwartz S. H. (1994). Values and personality. *Eur. J. Pers.* 8 163–181.

[B4] Bobowik M. Basabe N. Páez D. Jiménez A. Bilbao M. Á (2011). Personal values and well-being among europeans, spanish natives and immigrants to Spain: does the culture matter? *J. Happ. Stud.* 12 401–419. 10.1007/s10902-010-9202-1

[B5] Boehm C. (2001). *Hierarchy in the Forest: The Evolution of Egalitarian Behavior.* Cambridge, MA: Harvard University Press.

[B6] Bradshaw E. L. Sahdra B. K. Calvo R. A. Mrvaljevich A. Ryan R. M. (2018a). Users’ intrinsic goals linked to alcohol dependence risk level and engagement with a health promotion website (Hello Sunday Morning): observational study. *J. Med. Internet Res. Mental Health* 5:e10022. 10.2196/10022 30348624 PMC6231871

[B7] Bradshaw E. L. Sahdra B. K. Ciarrochi J. Parker P. D. Martos T. Ryan R. M. (2018b). A configural approach to aspirations: the social breadth of aspiration profiles predicts well-being over and above the intrinsic and extrinsic aspirations that comprise the profiles. Manuscript submitted for publication. 10.1037/pspp0000374 33271051

[B8] Brambilla M. Assor A. Manzi C. Regalia C. (2015). Autonomous versus controlled religiosity: family and group antecedents. *Int. J. Psychol. Relig.* 25 193–210. 10.1080/10508619.2014.888902

[B9] Browne M. W. (1992). Circumplex models for correlation matrices. *Psychometrika* 57 469–497. 10.1007/bf02294416 10948981

[B10] Clark L. A. Watson D. (1995). Constructing validity: basic issues in objective scale development. *Psychol. Assess.* 7 309–319. 10.1037/1040-3590.7.3.309 PMC6754793 30896212

[B11] Cohen S. Williamson G. (1988). “Perceived stress in a probability sample of the United States,” in *The social psychology of health: Claremont Symposium on applied social psychology*, eds Spacapam S. Oskamp S. (Newbury Park, CA: Sage), 31–67.

[B12] Csardi G. Nepusz T. (2006). The igraph software package for complex network research. *InterJ. Complex Syst.* 1695 1–9.

[B13] Daniels L. M. Haynes T. L. Stupnisky R. H. Perry R. P. Newall N. E. Pekrun R. (2008). Individual differences in achievement goals: a longitudinal study of cognitive, emotional, and achievement outcomes. *Contemp. Educ. Psychol.* 33 584–608. 10.1016/j.cedpsych.2007.08.002

[B14] Deci E. L. Ryan R. M. (2000). The “what” and “why” of goal pursuits: human needs and the self-determination of behavior. *Psychol. Inq.* 11 227–268. 10.1207/s15327965pli1104_01 27055568

[B15] Diener E. Emmons R. A. Larsen R. J. Griffin S. (1985). The satisfaction with life scale. *J. Pers. Assess.* 49 71–75. 16367493 10.1207/s15327752jpa4901_13

[B16] Diener E. Wirtz D. Tov W. Kim-Prieto C. Choi D. Oishi S. (2010). New well-being measures: short scales to assess flourishing and positive and negative feelings. *Soc. Indic. Res.* 97 143–156. 10.1007/s11205-009-9493-y

[B17] Eisenberger N. I. Lieberman M. D. Williams K. D. (2003). Does rejection hurt? An fMRI study of social exclusion. *Science* 302 290–292. 10.1126/science.1089134 14551436

[B18] Epskamp S. Borsboom D. Fried E. I. (2018). Estimating psychological networks and their accuracy: a tutorial paper. *Behav. Res. Methods* 50 195–212. 10.3758/s13428-017-0862-1 28342071 PMC5809547

[B19] Epskamp S. Cramer A. O. Waldorp L. J. Schmittmann V. D. Borsboom D. (2012). qgraph: network visualizations of relationships in psychometric data. *J. Stat. Softw.* 48 1–18. 10.18637/jss.v048.i04

[B20] Fodor E. M. Smith T. (1982). The power motive as an influence on group decision making. *J. Pers. Soc. Psychol.* 42 178–185. 10.1007/s11031-018-9687-1 29720775 PMC5915518

[B21] Friedman J. Hastie T. Tibshirani R. (2018). *glasso: Graphical Lasso: Estimation of Gaussian Graphical Models. R Package Version 1.10.*

[B22] Grassi M. Luccio R. Blas L. D. (2010). CircE: an R implementation of Browne’s circular stochastic process model. *Behav. Res. Methods* 42 55–73. 10.3758/BRM.42.1.55 20160286

[B23] Grouzet F. M. E. Kasser T. Ahuvia A. Dols J. M. F. Kim Y. Lau S. (2005). The structure of goal contents across 15 cultures. *J. Pers. Soc. Psychol.* 89 800–816. 10.1037/0022-3514.89.5.800 16351369

[B24] Joshanloo M. Ghaedi G. (2009). Value priorities as predictors of hedonic and eudaimonic aspects of well-being. *Pers. Indiv. Diff.* 47 294–298. 10.1016/j.paid.2009.03.016

[B25] Kasser T. Rosenblum K. L. Sameroff A. Deci E. L. Niemiec C. P. Ryan R. M. (2014). Changes in materialism, changes in psychological well-being: evidence from three longitudinal studies and an intervention experiment. *Motiv. Emot.* 38 1–22. 10.1007/s11031-013-9371-4

[B26] Kasser T. Ryan R. M. (1993). A dark side of the American dream: correlates of financial success as a central life aspiration. *J. Pers. Soc. Psychol.* 65 410–422. 10.1037//0022-3514.65.2.410 8366427

[B27] Kasser T. Ryan R. M. (1996). Further examining the American dream: differential correlates of intrinsic and extrinsic goals. *Pers. Soc. Psychol. Bull.* 22 280–287. 10.1177/0146167296223006

[B28] Kasser T. Ryan R. M. (2001). “Be careful what you wish for: optimal functioning and the relative attainment of intrinsic and extrinsic goals,” in *Life Goals and Well-Being: Towards a Positive Psychology of Human Striving*, eds Schmuck P. Sheldon K. M. (Seattle, DC: Hogrefe & Huber Publishers), 116–131.

[B29] Kenny D. A. Kaniskan B. McCoach D. B. (2015). The performance of RMSEA in models with small degrees of freedom. *Sociol. Methods Res.* 44 486–507. 10.1177/0049124114543236

[B30] Kerr N. L. Levine J. M. (2008). The detection of social exclusion: evolution and beyond. *Group Dyn. Theory Res. Pract.* 12 39–52. 10.1037/1089-2699.12.1.39 27540705

[B31] Lammers J. Stoker J. I. Rink F. Galinsky A. D. (2016). To have control over or to be free from others? The desire for power reflects a need for autonomy. *Pers. Soc. Psychol. Bull.* 42 498–512. 10.1177/0146167216634064 26984014

[B32] Legate N. De Haan C. R. Weinstein N. Ryan R. M. (2013). Hurting you hurts me too: the psychological costs of complying with ostracism. *Psychol. Sci.* 24 583–588. 10.1177/0956797612457951 23447557

[B33] Lekes N. Gingras I. Philippe F. L. Koestner R. Fang J. (2010). Parental autonomy-support, intrinsic life goals, and well-being among adolescents in China and North America. *J. Youth Adolesc.* 39 858–869. 10.1007/s10964-009-9451-7 19771500

[B34] Locke E. A. Latham G. P. (1990). *A Theory of Goal Setting & Task Performance.* Englewood Cliffs, NJ: Prentice-Hall, Inc.

[B35] Martela F. Ryan R. M. (2019). Distinguishing between basic psychological needs and basic wellness enhancers: the case of beneficence as a candidate psychological need. *Motiv. Emot.* (in press). 10.1007/s11031-019-09800-x

[B36] Martos T. Kezdy A. Horvath-Szabo K. (2011). Religious motivations for everyday goals: their religious context and potential consequences. *Motiv. Emot.* 35 75–88. 10.1007/s11031-010-9198-1

[B37] Martos T. Kopp M. S. (2012). Life goals and well-being: Does financial status matter? Evidence from a representative Hungarian sample. *Soc. Indic. Res.* 105 561–568. 10.1007/s11205-011-9788-7

[B38] Mason W. Suri S. (2012). Conducting behavioral research on Amazon’s mechanical turk. *Behav. Res. Methods* 44 1–23. 10.3758/s13428-011-0124-6 21717266

[B39] McClelland D. C. (1979). Inhibited power motivation and high blood pressure in men. *J. Abnorm. Psychol.* 88 182–190. 10.1037/0021-843X.88.2.182 447901

[B40] McClelland D. C. (1985). *Human Motivation.* Glenview, IL: Scott Foresman.

[B41] Mundfrom D. J. Shaw D. G. Ke T. L. (2005). Minimum sample size recommendations for conducting factor analyses. *Int. J. Test.* 5 159–168. 10.3109/09540261.2011.560143 21521091

[B42] Niemiec C. P. Ryan R. M. Deci E. L. (2009). The path taken: consequences of attaining intrinsic and extrinsic aspirations in post-college life. *J. Res. Pers.* 43 291–306. 10.1016/j.jrp.2008.09.001 20161160 PMC2736104

[B43] Nishimura T. Bradshaw E. L. Deci E. L. Ryan R. M. (2019). Satisfaction of basic psychological needs in an interdependence model of fathers’ and their children’s aspirations. Manuscript submitted for publication.

[B44] O’Connor B. P. (2000). SPSS and SAS programs for determining the number of components using parallel analysis and Velicer’s MAP test. *Behav. Res. Methods Instrum. Comp.* 32 396–402. 10.3758/bf03200807 11029811

[B45] Osborne J. W. Costello A. B. Kellow J. T. (2008). “Best practices in exploratory factor analysis,” in *Best Practices in Quantitative Methods*, ed. Osborne J. W. (Thousand Oaks, CA: SAGE Publications), 86–99. 10.4135/9781412995627.d8

[B46] R Core Team (2018). *R: A Language and Environment for Statistical Computing (Version 3.5.1).* Vienna: R Foundation for Statistical Computing.

[B47] Revelle W. (2018). *psych: Procedures for Personality and Psychological Research R Package Version 1.8.4.* Evanston, IL: Northwestern University.

[B48] Romero E. Gómez-Fraguela J. A. Villar P. (2012). Life aspirations, personality traits and subjective well-being in a Spanish sample. *Eur. J. Pers.* 26 45–55. 10.1002/per.815

[B49] Ryan R. M. Chirkov V. I. Little T. D. Sheldon K. M. Timoshina E. Deci E. L. (1999). The American dream in Russia: extrinsic aspirations and well-being in two cultures. *Pers. Soc. Psychol. Bull.* 25 1509–1524. 10.1177/01461672992510007

[B50] Ryan R. M. Deci E. L. (2000). The Darker and brighter sides of human existence: basic psychological needs as a unifying concept. *Psychol. Inq.* 11 319–338. 10.1207/s15327965pli1104_03

[B51] Ryan R. M. Deci E. L. (2017). *Self-Determination Theory: Basic Psychological Needs in Motivation, Development, and Wellness.* New York, NY: Guilford Press.

[B52] Ryan R. M. Frederick C. (1997). On energy, personality, and health: subjective vitality as a dynamic reflection of well-being. *J. Pers.* 65 529–565. 10.1111/j.1467-6494.1997.tb00326.x 9327588

[B53] Ryan R. M. Rigby S. King K. (1993). Two types of religious internalization and their relations to religious orientations and mental health. *J. Pers. Soc. Psychol.* 65 586–586. 8410653 10.1037//0022-3514.65.3.586

[B54] SabzehAra M. Ferguson Y. L. Sarafraz M. R. Mohammadi M. (2014). An investigation of the associations between contingent self-worth and aspirations among Iranian university students. *J. Soc. Psychol.* 154 59–73. 10.1080/00224545.2013.843501 24689337

[B55] Sagiv L. Schwartz S. H. (2000). Value priorities and subjective well-being: direct relations and congruity effects. *Eur. J. Soc. Psychol.* 30 177–198. 10.1002/(sici)1099-0992(200003/04)30:2<177::aid-ejsp982>3.0.co;2-z

[B56] Schwartz S. H. Cieciuch J. Vecchione M. Davidov E. Fischer R. Beierlein C. (2012). Refining the theory of basic individual values. *J. Pers. Soc. Psychol.* 103 663–688. 10.1037/a0029393 22823292

[B57] Sortheix F. M. Schwartz S. H. (2017). Values that underlie and undermine well-being: variability across Countries. *Eur. J. Pers.* 31 187–201. 10.1002/per.2096

[B58] Spitzer R. L. Kroenke K. Williams J. W. Löwe B. (2006). A brief measure for assessing generalized anxiety disorder: the gad-7. *Arch. Intern. Med.* 166 1092–1097. 16717171 10.1001/archinte.166.10.1092

[B59] Spoor J. Williams K. D. (2007). “The evolution of an ostracism detection system,” in *Evolution and the Social Mind: Evolutionary Psychology and Social Cognition*, eds Forgas J. P. Haselton M. von Hippel W. (New York, NY: Psychology Press), 279–292.

[B60] Steger M. F. Frazier P. Oishi S. Kaler M. (2006). The meaning in life questionnaire: assessing the presence of and search for meaning in life. *J. Counsel. Psychol.* 53 80–93. 10.1080/00223891.2013.765882 23406365

[B61] Tuominen-Soini H. Salmela-Aro K. Niemivirta M. (2012). Achievement goal orientations and academic well-being across the transition to upper secondary education. *Learn. Indiv. Diff.* 22 290–305. 10.1016/j.lindif.2012.01.002

[B62] Unanue W. Dittmar H. Vignoles V. L. Vansteenkiste M. (2014). Materialism and well-being in the UK and Chile: basic need satisfaction and basic need frustration as underlying psychological processes. *Eur. J. Pers.* 28 569–585.

[B63] Van Hiel A. Vansteenkiste M. (2009). Ambitions fulfilled? The effects of intrinsic and extrinsic goal attainment on older adults’ ego-integrity and death attitudes. *Int. J. Aging Hum. Dev.* 68 27–51. 10.2190/ag.68.1.b 19348103

[B64] Wesselmann E. D. Nairne J. S. Williams K. D. (2012). An evolutionary social psychological approach to studying the effects of ostracism. *J. Soc. Evol. Cult. Psychol.* 6 309–328. 10.1037/h0099249

[B65] Wickham H. (2009). *ggplot2: Elegant Graphics for Data Analysis.* New York, NY: Springer-Verlag.

[B66] Wickham H. François R. Henry L. Müller K. (2018). *dplyr: A Grammar of Data Manipulation. R Package Version 0.7.5.*

[B67] Wickham H. Henry L. (2018). *tidyr: Easily Tidy Data With ‘Spread()’ and ‘Gather()’ Functions. R Package Version 0.8.1.*

[B68] Williams K. D. (2009). “Ostracism: a temporal need-threat model,” in *Advances in Experimental Social Psychology*, Vol. 41 ed. Zanna M. (New York, NY: Academic Press), 275–314.

[B69] Winter D. G. John O. P. Stewart A. J. Klohnen E. C. Duncan L. E. (1998). Traits and motives: toward an integration of two traditions in personality research. *Psychol. Rev.* 105 230–250. 10.1037//0033-295x.105.2.230 9577238

